# JAK/STAT-Dependent Chimeric Antigen Receptor (CAR) Expression: A Design Benefiting From a Dual AND/OR Gate Aiming to Increase Specificity, Reduce Tumor Escape and Affect Tumor Microenvironment

**DOI:** 10.3389/fimmu.2021.638639

**Published:** 2021-06-09

**Authors:** Javad Khanali, Mohammadreza Azangou-Khyavy, Melika Boroomand-Saboor, Mobina Ghasemi, Hassan Niknejad

**Affiliations:** ^1^ School of Medicine, Shahid Beheshti University of Medical Sciences, Tehran, Iran; ^2^ Department of Pharmacology, School of Medicine, Shahid Beheshti University of Medical Sciences, Tehran, Iran

**Keywords:** cancer, adoptive immunotherapy, chimeric antigen receptors T cells, tumor escape and relapse, tumor microenvironment, cancer-associated fibroblasts, on-target/off-tumor toxicity, bispecific T cell engagers (BiTEs)

## Abstract

Recent advances in cancer immunotherapy have attracted great interest due to the natural capacity of the immune system to fight cancer. This field has been revolutionized by the advent of chimeric antigen receptor (CAR) T cell therapy that is utilizing an antigen recognition domain to redirect patients’ T cells to selectively attack cancer cells. CAR T cells are designed with antigen-binding moieties fused to signaling and co-stimulatory intracellular domains. Despite significant success in hematologic malignancies, CAR T cells encounter many obstacles for treating solid tumors due to tumor heterogeneity, treatment-associated toxicities, and immunosuppressive tumor microenvironment. Although the current strategies for enhancing CAR T cell efficacy and specificity are promising, they have their own limitations, making it necessary to develop new genetic engineering strategies. In this article, we have proposed a novel logic gate for recognizing tumor-associated antigens by employing intracellular JAK/STAT signaling pathway to enhance CAR T Cells potency and specificity. Moreover, this new-generation CAR T cell is empowered to secrete bispecific T cell engagers (BiTEs) against cancer-associated fibroblasts (CAFs) to diminish tumor metastasis and angiogenesis and increase T cell infiltration.

## Introduction

**Figure 1:**
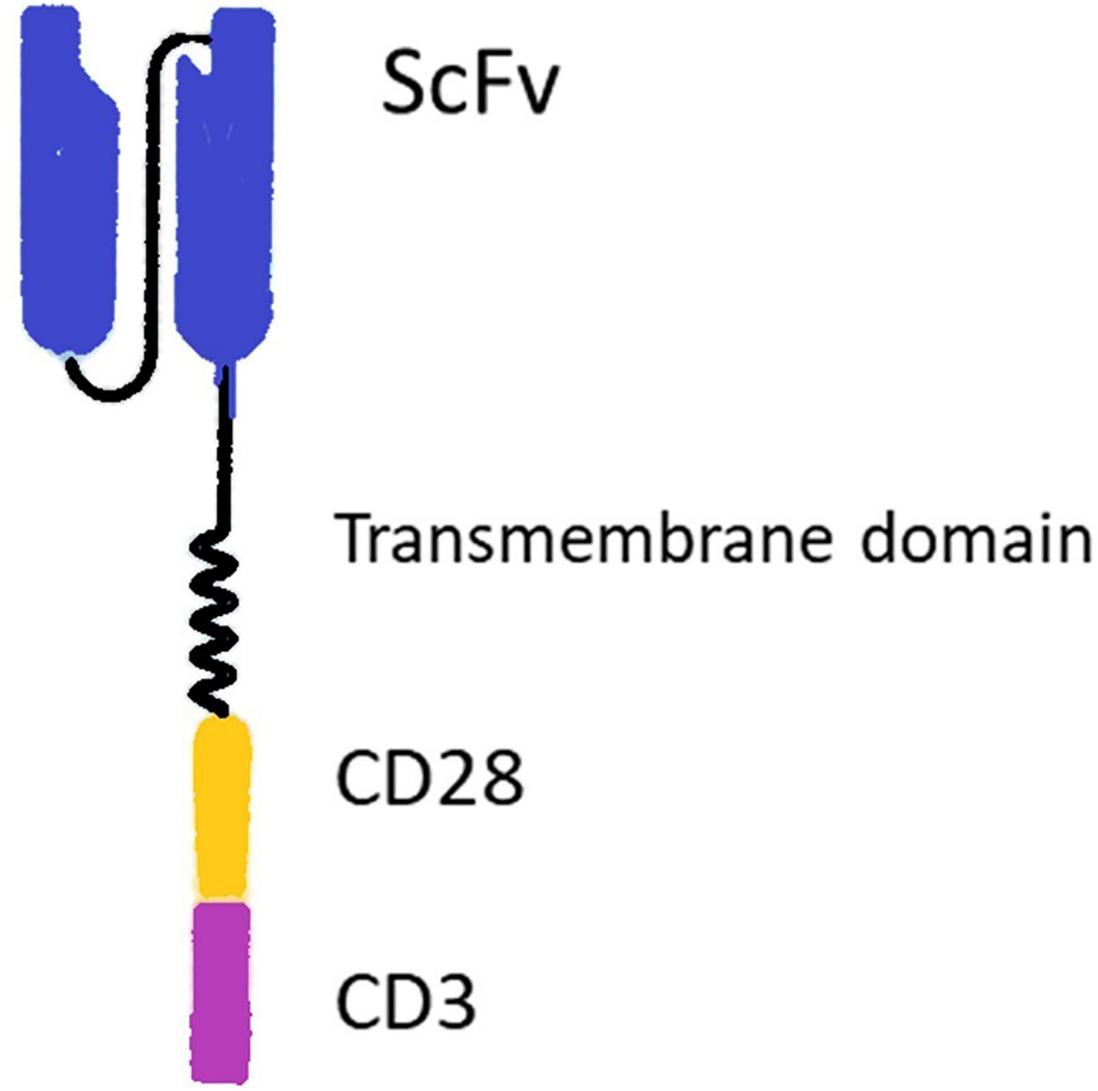
Structure of a chimeric antigen receptor (CAR). The CAR includes extracellular binding moiety (ScFv), a transmembrane domain, and intracellular signaling domains (i.e., usually CD3ζ chain of T cell receptor and CD28 co-stimulatory domain).

T cell-based immunotherapies have been revolutionized after the advent of chimeric antigen receptor T cells (CARs) (1), which are constructed through transfecting T cells by CAR genes. CARs consist of an extracellular antigen-binding moiety and intracellular T cell activating domain. The extracellular domain, named single-chain variable fragment (ScFv), is a fusion protein made up of the heavy (V_H_) and light chains (V_L_) of immunoglobulins. ScFv binds to tumor-associated antigens (TAAs). The intracellular signaling domain (i.e., CD3ζ chain of T cell receptor and usually CD28 and/or 4-1BB as co-stimulatory domain) stimulates cytotoxic T cell activity following TAA recognition (**Figure 1**).

ScFv could directly recognize TAAs. Thus, CAR T cells are able to detect tumor cells regardless of recognizing MHC molecules that are variably down-regulated in tumor cells to limit T cell-mediated cytotoxicity (2). Therefore, genetically engineering T cells to express CARs results in improved antigen-specific T cell cytotoxicity (3). This strategy combines the MHC-independence of mABs with the killing potential of T cells to confer tumor-specific cytotoxicity.

The remarkable success of CAR T cells in liquid malignancies has led to the FDA’s approval of these cells to treat non-Hodgkin lymphoma and refractory acute lymphoblastic leukemia (4). However, CAR T cell therapy has a variety of challenges in the treatment of solid tumors. First, a predetermined target antigen, which is supposed to be expressed on the malignant cell surface, could also be expressed on normal tissue cells. Therefore, the injected CAR T cells could harm normal tissues due to this problem, which is termed as on-target off-tumor toxicity (5). Moreover, CAR T cells are programmed to detect a specific antigen, whereas tumor cells express antigens heterogeneously. Heterogeneity of tumor cell surface antigens leads to tumor escape due to low-level expression of targeted antigens on some tumor cells. The third well-established challenge of CAR T cell therapy in solid tumors is the immunosuppressive effect of the tumor microenvironment. The tumor microenvironment includes a mixture of resident immune cells, fibroblasts, and immunosuppressive cytokines that interferes with the infiltration and function of CAR T cells (6).

To overcome these challenges in CAR T cell therapy, we propose a novel design of CAR T cells. In this hypothesis, the proposed CAR T cell retains features to address the discussed challenges. In brief, the CAR T cell contains a novel synthetic receptor that induces the intracellular signaling pathway followed by recognizing the tumor-associated antigen, which leads to the expression of two different CARs. This CAR T cell will be activated in the simultaneous presence of 2 out of 3 TAAs, which induces a novel logic gate to activate CAR T cells against tumor cells. Moreover, the CAR T cell will be hypothetically empowered to secrete the anti-CAF bispecific antibodies that can eliminate cancer-associated fibroblasts. This elimination will reduce tumor metastasis by inhibiting angiogenesis and enhancing T cell infiltration into the tumor microenvironment.

## Supporting Evidence

**Figure 2:**
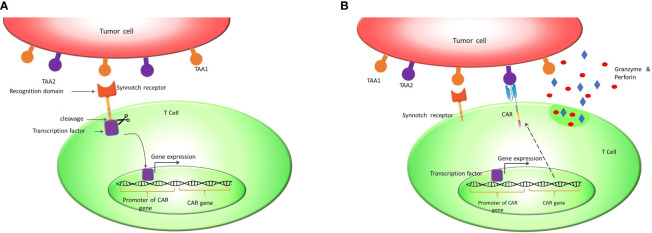
Synnotch receptor CAR T cells: Synnotch receptors are designed to pursue the AND logic gate to mitigate on-target/off-tumor toxicity. **(A)** These receptors release a synthetic chimeric transcription factor (GAL4-VP64) after recognizing tumor-associated antigen 1 (TAA1). **(B)** The transcription factor binds to the GAL4 responsive promoter and induces the expression of CAR against TAA2.

Since on-target/off-tumor toxicity is a matter of concern, an antigen that is not expressed within normal tissues would be an ideal target antigen for CAR T cell therapy. However, the vast majority of candidate antigens are also expressed on normal tissues at lower levels. There are several creative solutions to overcome this problem, including OFF-switch degradable CARs linked to degron tags or ON-switch split CARs responsive to drug, along with suicide genes and logic gates  (7, 8). One of the most studied solutions in this regard is implementing CAR T cells that follow AND logic in recognizing target antigens (9–11). These ‘AND gated’ CAR T cells need recognition of two targeted antigens on the cell surface for activation and cytotoxicity (11). Since the expression of two TAAs simultaneously on the surface of normal cells is less probable, “AND logic” can mitigate the risk of on-target/off-tumor toxicity. This strategy was previously applied in CAR T cells by implementing a synthetic notch receptor (synNotch receptor). SynNotch receptor is designed to have ScFv domain as an extracellular domain, a transmembrane domain, and a transcription factor as an intracellular domain. After recognizing the first antigen by ScFv, S2 and S3 cleavage sites primarily autoinhibited by a negative regulatory region (NRR) are exposed. Antigen binding causes NRR conformation change, which signals the transmembrane domain (TMD). Subsequent to these changes, S2 and S3 sites are exposed to A Disintegrin And Metalloproteinase (ADAM) and γ-secretas. These enzymes cleave S2 and S3 sites, and the transcription factor is released subsequently (12, 13). Afterward, the transcription factor binds to its specific promoter in the vector and drives the expression of the CAR receptor against the second antigen (14, 15). Accordingly, the presence of the second tumor-associated antigen activates the CAR receptor and initiates cytotoxic reactions. Therefore, the presence of two tumor-associated antigens on the tumor cell surface is critical for cytotoxic reactions in this design, which practically increase specificity and decrease on-target/off-tumor toxicity (**Figure 2**).

The second problem in treating solid tumors by CAR T cells is tumor cell heterogeneity. An approach to solve this problem is using ‘OR gated’ CAR T cells, which are able to target various antigens on different tumor cells to provide better killing coverage (9). These T cells are designed to express two or more CARs against different TAAs on a single cell. Since each of the CAR’s intracellular domains consists of both CD3 and co-stimulatory domains, recognizing each of the TAAs would be enough for CAR T cell activation. This strategy has been evaluated in pre-clinical studies, where “OR gated” CAR T cells have targeted either CD19 or CD123 TAAs to treat lymphoma (16). Similarly, trivalent CAR T cells have been directed to HER2, IL13Rα2, and EphA2 antigens to target glioblastoma. This trivalent CAR T cell was able to eradicate tumor cells in nearly all patient models (17).

Even though “AND” and “OR” strategies are promising, each one has its limitations, highlighting the need to develop genetically engineered control systems to overcome these challenges. “AND gate” strategy increases the probability of tumor escape because of reducing killing coverage. On the other side, the OR gate approach can increase the probability of on-target/off-tumor toxicity. To bypass this challenge, the balance of killing coverage and safety is an important hallmark for improving CAR T cell therapy (18, 19).

The third problem of CAR T cell therapy for solid tumors is the immune-suppressive tumor microenvironment that one of its immunosuppressive and anti-infiltrative components is cancer-associated fibroblast (CAF). CAFs are stromal cells affecting tumor cells and their microenvironment in a pro-tumorigenic manner. The activity of CAFs results in a more aggressive tumor formation, progression, and metastasis (20). Thus, killing these cells would be promising in enhancing CAR T cell therapy efficacy. It is also worth noting that fibroblast activation protein-a (FAP) is highly overexpressed on cancer-associated fibroblasts, and it has been shown that targeting cancer-associated fibroblasts by anti-FAP CAR T cells inhibits tumor growth and augments host immunity against tumor (21).

In addition to developing anti-FAP CARs, there are several other methods of directing T cells toward target cells, among which antibody-derived molecules are growing rapidly. In this regard, Bispecific T cell Engagers (BiTEs) which were first approved by the FDA for the treatment of refractory acute lymphoblastic leukemia in 2014 (22), can facilitate the T cell and target cell interaction (23). BiTEs are a class of bispecific antibodies that could be constructed by attaching two ScFv domains by a linker. In adoptive cancer immunotherapy, these agents are constructed to be directed against the CD3 complex on the T cell’s surface and an antigen on the target cell. BiTEs redirect the cytotoxic activity of T cells against the target cells in a non-MHC-dependent fashion (24). Likewise, studies have revealed that employing BiTEs to target CAFs could be effective. Accordingly, injected anti-FAP BiTE and its infiltration to tumor microenvironment increase the intra-tumoral accumulation of T cells and cause cytotoxicity in cancer-associated fibroblasts (25). Since it has been shown that CAR T cells could successfully secrete BiTEs (26), producing anti-FAP BiTEs by CAR T cells locally in the tumor microenvironment would be on the horizon, which will be discussed later in this paper.

## Hypothesis

**Figure 3:**
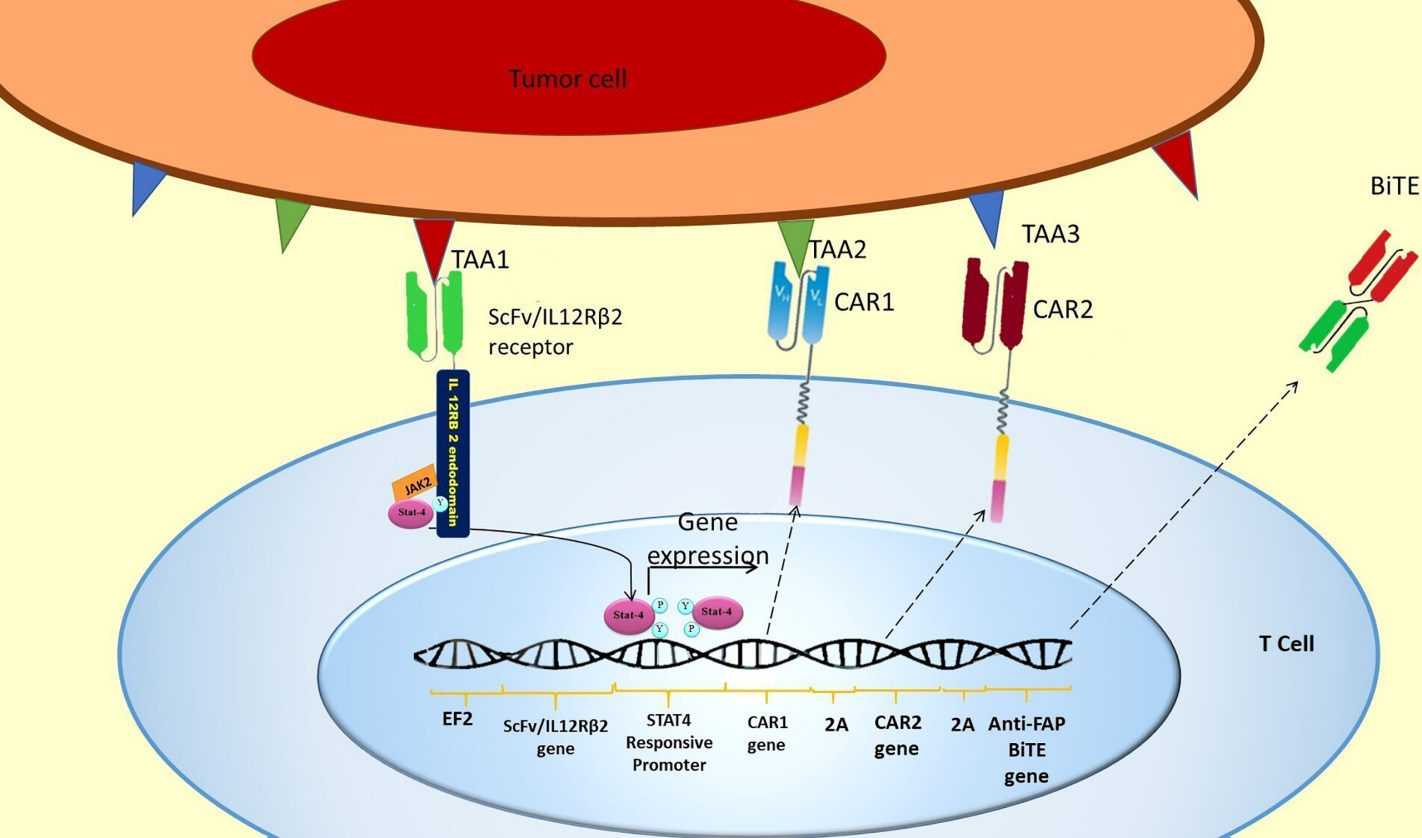
SCFV/IL-12Rβ2 receptor induces CAR genes expression. The hypothetically designed cells express ScFv/IL-12Rβ2 receptor constitutively before injection. The ScFv/IL-12Rβ2 receptor consists of an extracellular domain against tumor-associated antigen 1 and the intracellular domain of β2 subunit of the human IL-12 receptor. After injection and TAA1 recognition by ScFv/IL-12Rβ2 receptor, JAK2 activates and creates a docking site for STAT4 attachment to ScFv/IL-12Rβ2 receptor endodomain. This attachment will activate the JAK2/STAT4 signaling pathway and STAT4 mediated gene expression after TAA1 recognition (27, 28). Based on our design, two different CAR receptors and anti-FAP BiTE genes are downstream of STAT4 responsive promoter; therefore, these genes will be expressed in consequence of TAA1 presence. Since a 2A ribosomal skip sequence separates these genes, they will be expressed simultaneously after STAT4 binding to the promoter. After CAR 1 and CAR 2 expression, they could recognize TAA2 and TAA3, respectively, and subsequently activate the CAR T cell.

An AND logic gate could be designed using internal T cell signaling pathways. For this purpose, JAK2/STAT4 signaling could be hijacked for the expression of our CAR genes. To put this in practice, a novel chimeric ScFv/IL-12Rβ2 receptor is proposed, which has an extracellular ScFv domain against the first antigen and the intracellular domain of β2 subunit of the human IL-12 receptor (**Figure 3**). The intracellular domain activates JAK2/STAT4 as a part of the IL-12 natural signaling pathway in T cells (29). By inserting a STAT4 responsive promoter in the vector, it would be possible to induce the expression of CAR genes and other genes of interest by putting them downstream of the mentioned promoter (**Figure 3**).

In this design, two different CAR genes are inserted downstream of the STAT responsive promoter. Therefore, the two different CAR receptors would be co-expressed after the activation of STAT4 and binding to its promoter (**Figure 3**). Since each of the expressed CARs has CD3 and CD28 intracellular domains, each one of them can activate the CAR T cell in response to the recognition of their specific antigen (TAA2 and TAA3) (**Figure 3**).

**Figure 4:**
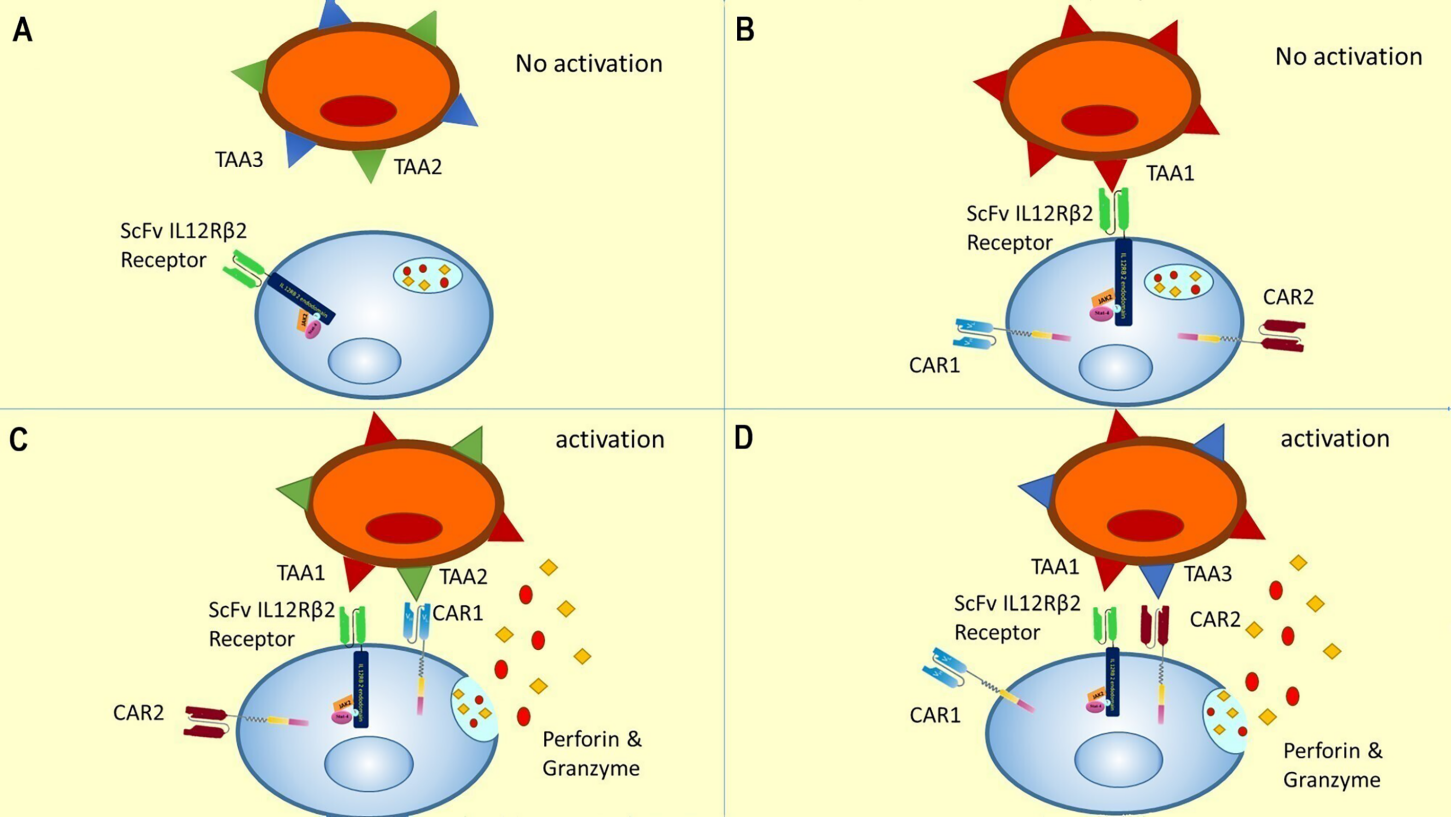
A novel gate for CAR T cells activation encountering different TAA profiles (four out of eight are shown). **(A)** In the absence of TAA1, there is no initiation of JAK/STAT pathway, no expression of CAR1 and CAR2, and accordingly no activation of CAR T cell. This condition could be representative of antigen profiles 5 to 8 in **Table 1**. **(B)** Recognition of TAA1 in the absence of TAA2 or TAA3 leads to internal pathway activation and CAR receptor expression, but the CAR T cells could not be activated without recognizing TAA2 or TAA3. This condition is representative of antigen profile 4 in **Table 1**. **(C)** According to our proposed novel logic gate, the simultaneous presence of TAA1 and TAA2 leads to the activation of the JAK/STAT pathway due to recognition of TAA1 and induction of cytotoxic reactions due to recognition of TAA2. This condition was represented in the second antigen profile in **Table 1**. **(D)** Concurrent presence of TAA1 and TAA3 and recognition of them by the CAR T cell leads to JAK/STAT activation and cytotoxic response. This condition is representative of the third antigen profile in **Table 1**. It can be easily concluded that the concurrent presence of all three antigens could lead to a cytotoxic response by activating CAR1 and CAR2, and this was represented in the first antigen profile in **Table 1**.

This order in CAR expression enables CAR T cells to initiate cytotoxic reactions when the TAA1 (which is recognized by ScFv/IL-12Rβ2 receptor) is expressed simultaneously with the TAA2 (which is recognized by CAR1) or TAA3 (which is recognized by CAR2) on the tumor cell surface. In other words, this gate is a novel 3-input/1-output logic gate in CAR T cell therapy that the inputs are TAAs, and the output is cytotoxicity. The CAR T cell could only show cytotoxicity in response to specific TAA profiles (shown in **Table 1** and **Figure 4**).

**Table 1:** Eight possible TAA profiles of three antigens and expected CAR T cell responses toward them.

	TAA1 (recognized by ScFv/IL-12RB2)	TAA2 (recognized by CAR1)	TAA3 (recognized by CAR2)	Response (cytotoxicity)
**1**	+	**+**	**+**	**+**
**2**	+	**+**	**-**	**+**
**3**	+	**-**	**+**	**+**
**4**	+	**-**	**-**	**-**
**5**	-	**+**	**+**	**-**
**6**	-	**+**	**-**	**-**
**7**	-	**-**	**+**	**-**
**8**	-	**-**	**-**	**-**

As shown in **Table 1**, in the presence of the TAA1, two CARs will be expressed against TAA2 and TAA3. Therefore, the TAA1 and TAA2 positive antigen profile or TAA1 and TAA3 positive antigen profile will initiate the cytotoxicity response. Thus, this CAR T cell would be specific to tumor cells due to recognizing two TAA simultaneously and would have better killing coverage than conventional ‘AND gated’ CAR T cells (**Figure 4**).

**Figure 5:**
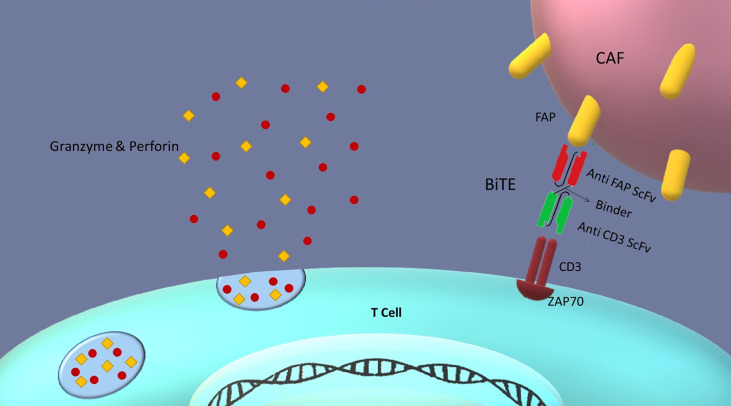
BiTEs role in the tumor microenvironment: Bispecific T cell engagers (BiTEs) are fusion proteins consisting of two ScFvs linked together. One of the ScFvs binds to the T cells CD3 marker and the other to an antigen on the targeted cell. Thus, they can establish a link between T cells and target cells in an MHC-independent manner. In our design, CAR T cells secrete BiTEs against FAP antigen on cancer-associated fibroblasts (CAFs); therefore, CAR T cells can be redirected against tumor-suppressive microenvironment by killing cancer-associated fibroblasts. This redirection could be favorable for cancer therapy because of reducing tumor progression and metastasis, and angiogenesis.

Besides, it is hypothesized that with the insertion of anti-FAP BiTE gene downstream of STAT4 responsive promoter, FAP antigen could be locally targeted on CAFs surface in the tumor microenvironment. This design enables our CAR T cells to secrete BiTEs in addition to the CAR expression in response to the STAT activation. BiTEs potently activate T cells without an apparent need for co-stimulatory domains (i.e., cd28.4-1bb) and can lead to a complete target cell (i.e., fibroblast) elimination (23). Therefore, targeting FAP-positive cancer-associated fibroblasts with anti-FAP BiTEs secreted from CAR T cells would be within the realm of possibility (**Figure 5**). Moreover, one of the advantages of the secreted BiTEs is that they can engage naive T cells with cancer-associated fibroblasts as well as CAR T cells.

## Evaluation of the Hypothesis

This study aims to design a new CAR T cell therapy strategy for increasing specificity, reducing tumor escape, and modulating tumor microenvironment for solid malignancies. The design consists of three receptors and a Bi-specific T cell Engager (BiTE).

### Preparation, Expansion, and Transduction of CAR T Cells

After negative selection, primary CD4+ and CD8+ T cells will be isolated from donor blood. Lentivirus will be produced through transfecting human embryonic kidney (HEK) cells with the transgene expression vector and packaging plasmids (30). The detailed processes of pre-transduction expansion, T cell transduction, and post-transduction expansion are based on the previous study (31).

### *In Vitro* Studies

#### Synthetic Receptor Expression and Function Assay

The Expression of ScFv/IL-12Rβ2 at mRNA and protein level could be measured *via* RT PCR and western blot, respectively. TAA1 positive cell lines will stimulate synthetic receptors expressed on the primary T cells surface. STAT4 phosphorylation and AKT phosphorylation, which are downstream of IL-12Rβ activation, could be measured *via* intracellular flow cytometry. Likewise, IFN gamma production could be measured *via* the ELISA technique (32).

As the STAT4 signaling pathway plays an important role in cell survival through Bcl-2, Bcl-XL, and MCL1 anti-apoptotic proteins, the expression of the mentioned anti-apoptotic proteins in the cells at mRNA and protein level could be measured before and after synthetic receptor activation *via* RT PCR and western blot, respectively (33).

#### CAR1 and CAR2 Gene Expression and Function Assay

Expression of CAR1 and CAR2 could be measured at mRNA and protein level *via* RT-PCR and flow cytometry (15), respectively. MTT and Chromium-51 cytotoxicity assays should be implemented to evaluate CAR1 and CAR2 cytotoxicity (33).

#### Anti-FAP BiTE Cytotoxicity Against FAP^+^ Cell Lines

BiTE gene expression could be measured at mRNA and protein level *via* RT-PCR and western blot (26). To obtain CAFs expressing FAP antigen, they should be isolated from the tumor microenvironment (34). To measure the BiTE induced cytotoxicity against CAFs, the fibroblasts could be labeled with chromium-51 and exposed to BiTEs.

### *In Vivo* Studies

#### CAR T Cell Injection Effects on Tumor

The genetically identical mouse tumor models will be formed to analyze the *in vivo* effects of CAR T cells on the tumor. Mice will be inoculated with 10^5^ tumor cells that express eight different antigen profiles mentioned in **Table 1**. The cells would be inoculated subcutaneously, and tumor volume will be measured using a caliper (34). After seven days, 10^5^ CAR T cells will be injected into the animal models. Tumor growth will be controlled post-inoculation every five days for 70 days. Besides, serum cytokine levels (e.g., IFN-γ) will be measured by the ELISA technique.

#### BiTE Production Effects on CAFs

Mice will be inoculated with 10^5^ TAA1 positive tumor cells and FAP positive CAFs. After seven days, 10^5^ CAR T cells would be injected into animal models. Since antigens 2 and 3 are absent in the used cell line, CAR1 and CAR2 will not be activated. Therefore, it is expected that the probable reduction in tumor size would be resultant of the release of BiTE from CAR T cells. Inoculated mice without CAR T cell injection could be used as the control group. Tumor growth would be controlled post-inoculation every five days for 70 days. Then, mice would be anesthetized, and the tumor would be removed from the mice, and tumor weight, VEGF, matrix metalloproteinase, and TGF β would be measured in tumor lysate by the ELISA technique in both groups. Since the fibroblasts are a producer of these cytokines in the tumor microenvironment, it is expected that these factors would be reduced in the test group as a result of fibroblasts killing.

## Discussion

The investigation of novel immunotherapeutic approaches has led to the development of CAR T cell therapy. However, CAR T cell-based therapies encounter different challenges. As mentioned above, challenges in CAR T cell therapy against solid tumors are due to the heterogenic expression of antigens on tumor cells surface and the probability of inadvertent targeting of non-tumoral cells. Furthermore, tumor cells can govern micro-environmental cells to alter the microenvironment and increase their survival against the host immune system. Here, we suggest a novel strategy to overcome these challenges in solid tumor treatments by CAR T cell therapy. Despite the previous studies that solely applied “AND” and “OR” logic gates in CAR T cell therapy, we think the combination of these two strategies is a more promising candidate for CAR T cell therapy of solid tumors. However, each of these gates can be rationally utilized based on the antigenic profile of the target tumor. AND gate is best suitable for the condition in which the target tumor expresses two non-specific antigens on a large proportion of tumor cells. Therefore, the non-specificity of the target antigens is fairly compensated by AND gate. On the other hand, OR gate is best suitable for the condition in which the target tumor expresses two specific antigens on a low proportion of tumor cells. Hence, the OR gate overcomes the challenge of proportionally low expression of the target antigens. Alternatively, the proposed dual logic gate here would be applicable for the condition in which the tumor highly expresses one non-specific antigen (TAA1) along with two other antigens that can even be non-specific and slightly expressed (TAA2 and TAA3).

**Figure 6:**
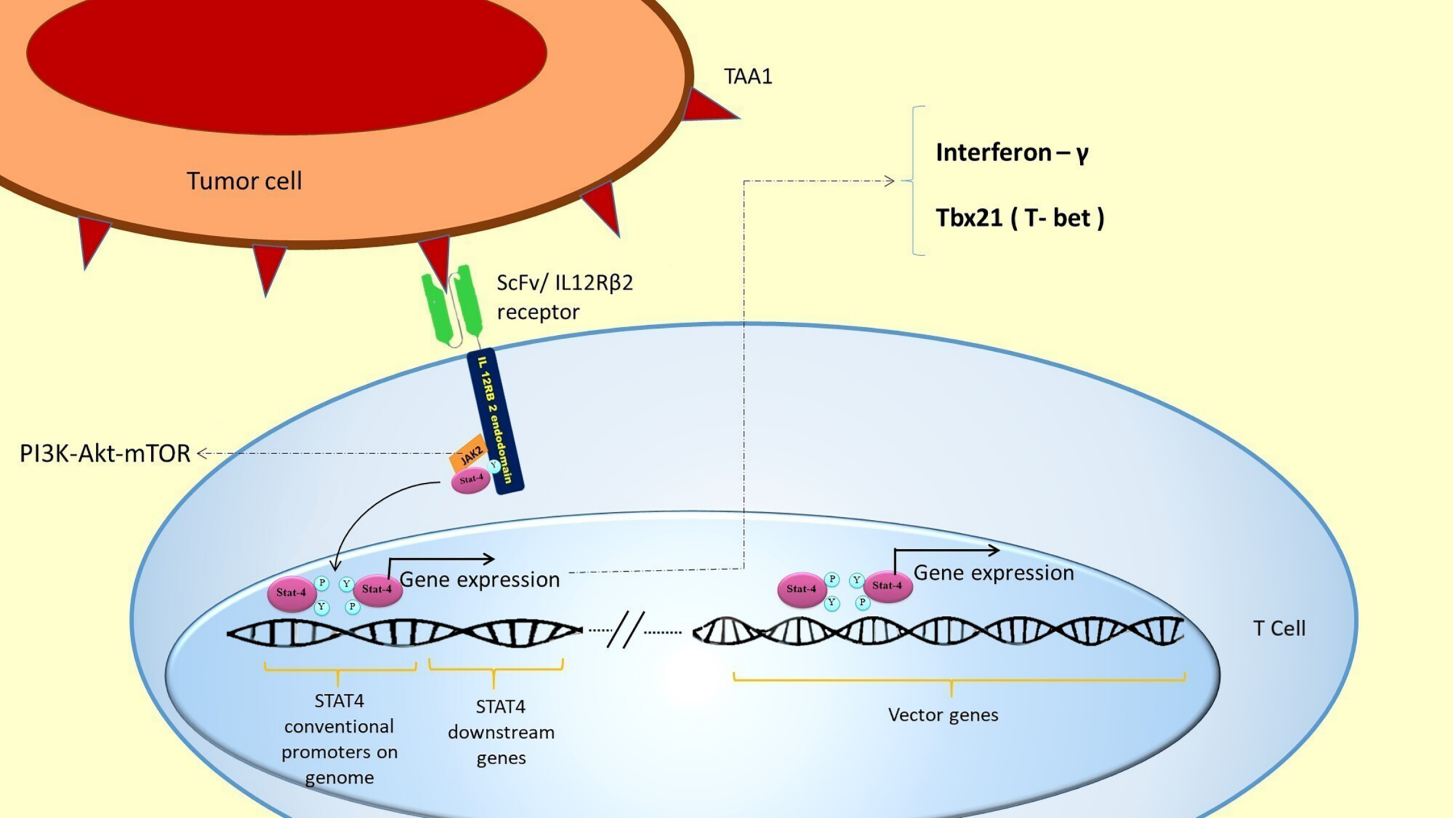
STAT4 activation and the expression of its downstream genes in the genome: TAA1 recognition by ScFv/IL-12Rβ receptor leads to STAT4 activation. Activated STAT4 binds to its conventional promoters in the T cell genome. This binding automatically expresses favorable genes in addition to expressing the inserted vector genes. STAT 4 conventional genes include IFN-γ and Tbx21 (T-bet), which induces TH1 phenotype and cytotoxicity in T cells (39). This expression could potentiate T cells’ proliferation and survival and attenuate inflammatory processes in the tumor area. JAK2 also activates PI3K/AKT/mTOR cell survival signaling pathway, which plays a pivotal role in T cell proliferation, survival, and protein synthesis.

As described above, the Synnotch system utilized a synthetic chimeric transcription factor and its responsive promoter to initiate the CAR gene expression (31). In contrast, we proposed the JAK2/STAT4 pathway to express two CAR receptors. Superior to Synnotch, there are no potentially immunogenic elements (GAL4-VP64) to express CARs (18). Besides, the designed T cells might be potentiated through activation of the IL-12 downstream signaling pathway. IL-12 receptors are composed of two subunits—IL-12Rβ1 and IL-12Rβ2. The IL-12Rβ1 is the shared receptor chain of the IL-12 and IL-23 receptor signaling complexes. IL-12Rβ1 activates TYK2 and STAT3, whereas IL-12Rβ2 activates JAK2/STAT4 signaling pathway (35). JAK2 is the first component of the JAK2/PI3K/AKT/mTOR cell survival signaling cascade that plays a pivotal role in T cell proliferation, survival, and protein synthesis (36, 37). Moreover, STAT4 induces the production of inflammatory cytokines such as IL-12 and IFN-γ, which strengthen T helper 1 and attenuate T helper 2 cell responses in the immune system. Since T helper 1 phenotype is correlated with inflammatory cytokine production such as IL-12 and IFNγ (which are helpful in cancer therapy), it would be favorable to intensify this phenotype. Also, the survival of T cells will be enhanced because of the proposed IL-12/STAT4 cascade. It is well known that IL-12/STAT4 signaling increases anti-apoptotic gene expression and decreases the expression of pro-apoptotic genes in T cells (38) (**Figure 6**). A study showed that using engineered NK92 cells to specifically release IL-12 at tumor sites has been demonstrated to increase the antitumor effects of CAR T cells. The study suggests antigen-directed IL-12 expression in the tumor microenvironment as a safe approach to enhance the clinical outcome of CAR-T cell therapy (40). Besides, STAT4 acts as a transcription factor for a core subset of genes and interacts with over 4000 genes through distinct binding motifs (41). Therefore, it seems that this transcription factor and its selected responsive promoter would be robust enough to drive CAR gene expression.

In the last part of our hypothesized strategy in treating solid tumors, the secretion of BiTEs from CAR T cells is proposed to alter the tumor microenvironment to enhance our system efficacy. Since fibroblasts can be governed by tumor cells and form CAFs, these cells have been chosen as the target for secreted BiTEs. CAFs affect tumor cells through paracrine signaling; they secrete CXCL12 (42), TGFβs (42), FGFs (43), periostin (POSTN) (44), and TN-C (45). These growth factors and cytokines increase tumor progression and metastasis and enhance tumor cell resistance to therapy. Furthermore, CAFs secrete matrix metalloproteinases (MMPs), a group of enzymes responsible for the extracellular matrix (ECM) degradation, and accordingly, tumor invasion and metastasis (46). In addition to remodeling the ECM by MMPs, CAFs secrete VEGF, FGF, and IL6 to remodel the tumor vasculature (47) and induce angiogenesis that is pivotal for tumor growth and metastasis (48). The other impact of CAFs on the tumor microenvironment is modulating pro-tumorigenic inflammation by secreting IL-1, IL-6, TNFα, TGFβs, SDF-1, and MCP-1 (47, 49). Hence, directing BiTEs towards CAFs would interfere with the impacts mentioned above. Finally, this targeting induces inflammation in the tumor microenvironment and probably could cause indirect killing of tumor cells (9). However, one of the concerns associated with the BiTEs is the off-target toxicity due to the engaging of native T cells’ CD3 antigen and FAP on non-tumor fibroblasts. Nevertheless, antigen-directed BiTE secretion locally in the tumor microenvironment using oncolytic viruses was demonstrated to cause minimized off-target toxicity (50). Besides, producing BiTE-secreting CAR T cells which secrete BiTEs locally have already been tested. In the study, antigens that entailed the risk of off-target toxicity in the case of systemic administration (i.e., EGFR) were successfully targeted locally in the tumor microenvironment (26). Therefore, the local secretion of BiTEs from CAR T cells in the proposed system might as well reduce the chance of potential off-target effects.

It is also worth noting that the interaction of CARs and TCRs might be concerning. However, they are less likely to share the same immunological synapses due to a number of reasons (51, 52). First, the immunological synapse of TCR is systematic (i.e., “bull’s eye” structure), whereas CAR immunological synapse is disorganized. Indeed, fewer interactions are needed for the formation of CAR immunological synapse, which results in higher affinity and shorter time for the formation of a functioning synapse. Second, TCR antigen recognition is MHC dependent while CAR antigen recognition is not. Third, studies have revealed that the signaling machinery downstream to the CAR is significantly different from TCR, despite common proteins in both pathways. Hence, knocking out the TCR on CAR T cells is not only unnecessary, but also it might have an intruding effect on the longevity of the anti-tumor response (53).

To the best of our knowledge, no studies have used intracellular pathways to induce CAR expression in a TAA-dependent manner. Likewise, the hypothesized logic gate for recognizing TAAs and anti-FAP BiTEs secreted from CAR T Cells is proposed for the first time. Based on CAR T cell therapy’s promising results, it is critical to investigate the hypothesized strategy in future studies.

## Limitations

Some limitations may interfere with our design’s efficacy. Most importantly, using an internal signaling pathway and its constitutive activation in T cells might interfere with the CARs expression. Despite the mentioned advantages of JAK/STAT signaling activation, this signaling is a common pathway among various cytokines that all could interfere with the desired gene expression (36). However, choosing IL-12Rβ2/JAK2/STAT4 signaling pathway in the design has the advantage of theoretically low inadvertent activation by the other cytokines. It has been shown that STAT4 is activated as a part of IL-12 and IL-23 pathways (35). Whereas other STAT family transcription factors are involved in many cytokine pathways, and this could disturb the considered “AND” gate by inappropriate CAR antigens expression (36). Although, it seems that IL-12-induced STAT4 activation may be concerning as IL-12 itself could cause inadvertent expression of CAR receptors. On the plus side, IL-12 is expected to be present in inflammatory regions such as the tumor microenvironment (27, 54). Therefore, IL-12 mediated CAR expression could be desirable, and it could be considered another tumor-specific condition to express CAR receptors. Further experiments have to be done to assess the risk of probable “on-target off-tumor toxicity” in the presence of various doses of IL-12 in extracellular fluid.

Despite these concerns, there are solutions to reduce the risk of this inadvertent expression of CAR receptors and the potential off-target effects. In addition to local infusion, suicide genes could be inserted into the vector. As a result, CAR T cell-mediated cytotoxicity could be blocked whenever the treatment is finished or off-target effects are detected. Another solution is to decrease the STAT4 promoter’s affinity to STAT4, which aims to ascertain that high levels of STAT4 are needed to express CAR receptors. Another rationale strategy can be using synthetic biology tools such as ribozymes, riboregulators, and tetracycline-responsive promoters to generate drug-inducible CAR T cells. Besides, Cre expression or release downstream of synthetic receptor activation could be considered. Adding LoxP sites flanked to the CAR genes and STAT-responsive promoter can strengthen the AND gate and hinder IL-12-induced T cell activation. However, if Cre-mediated CAR expression in a STAT-independent manner is considered the gate, it would result in a continuous CAR expression after TAA1 recognition that increases the probability of off-target effects (55, 56).

Furthermore, assessing two kinetics is important to determine the efficacy of the proposed gate. First, the time course of T cell activation upon stimulation with a dual-antigen (e.g., TAA1 and TAA2) tumor cell line is important to determine the half-life for T cell activation. This half-life was reported to be approximately 13 hours in the synNotch system consisted of 6 hours for CAR expression and 6 hours for subsequent T cell activation (31). In another study with the objective of CAR-mediated STAT activation, the time interval needed for STAT3 and STAT5 phosphorylation by IL-2Rβ was determined to be 2 hours. Therefore, on the premise that IL-12 Rβ2 functions similarly, and T cell activation subsequent to CAR expression would take 6 hours as in the synNotch system, the half-life for T cell activation in the proposed system is anticipated to be 8 hours plus the time needed for STAT4 mediated CAR expression.

The second important kinetic in this regard is the decay kinetic of synthetic receptor-induced CAR expression. The importance of this kinetic is due to the concern which was also aroused in the synNotch/CAR T cells termed as “priming” of CAR T cells. CAR T cells may engage with TAA1, which signals the expression of the CARs against other TAAs. These “primed” T cells could migrate to the other parts of the body and kill single antigen bystander tissues, which could disrupt the “AND” logic gate. However, previous studies have not shown on-target off-tumor toxicity due to the “priming” effect of synNotch/CARs in in-vivo models. This lack of toxicity could result from the kinetics of removal of CAR receptors from the cells’ surface. Indeed, CAR expression decay occurred in 8 hours, which was faster than the time needed for T cells to migrate out of the priming tumor and cause inadvertent cytotoxicity in a different immunological synapse (14). Since the proposed CAR expression mechanism here has not been studied yet, this system’s expression and removal kinetics is not confidently predictable and have to be evaluated in future experiments.

## Conclusions

Expressing CARs and secreting BiTEs by the suggested novel logic gate using the IL-12 natural signaling pathway could boost cancer treatment by CAR T cells due to increasing specificity, decreasing tumor escape, and increasing CAR T cells survival and cytotoxic potency as well as modulating the tumor microenvironment.

## Data Availability Statement

The original contributions presented in the study are included in the article/supplementary material. Further inquiries can be directed to the corresponding author.

## Author Contributions

JK, MA-K, MB-S, and MG conceived the idea and wrote the manuscript. HN assisted in developing the idea and revised the manuscript. All authors contributed to the article and approved the submitted version.

## Conflict of Interest

The authors declare that the research was conducted in the absence of any commercial or financial relationships that could be construed as a potential conflict of interest.

## References

[1] Li D Li X Zhou W-L Huang Y Liang X Jiang L. Genetically Engineered T Cells for Cancer Immunotherapy. Sig Transduct Target Ther (2019) 4(35):1–17. 10.1038/s41392-019-0070-9 PMC6799837 31637014

[2] Morrison BJ Steel JC Morris JC. Reduction of MHC-I Expression Limits T-Lymphocyte-Mediated Killing of Cancer-Initiating Cells. BMC Cancer (2018) 18(1):469. 10.1186/s12885-018-4389-3 29699516 PMC5918869

[3] Fesnak AD June CH Levine BL. Engineered T Cells: The Promise and Challenges of Cancer Immunotherapy. Nat Rev Cancer (2016) 16(9):566–81. 10.1038/nrc.2016.97 PMC5543811 27550819

[4] US Food & Drug Administratation. FDA Approves CAR-T Cell Therapy to Treat Adults With Certain Types of Large B Cell Lymphoma. USA: FDA (2017). Available at: https://www.fda.gov/newsevents/newsroom/pressannouncements/ucm581216.htm.

[5] Morgan RA Yang JC Kitano M Dudley ME Laurencot CM Rosenberg SA. Case Report of a Serious Adverse Event Following the Administration of T Cells Transduced With a Chimeric Antigen Receptor Recognizing ERBB2. Mol Ther J Am Soc Gene Ther (2010) 18(4):843–51. 10.1038/mt.2010.24 PMC2862534 20179677

[6] Newick K Moon E Albelda SM. Chimeric Antigen Receptor T-Cell Therapy for Solid Tumors. Mol Ther Oncolytics (2016) 3:16006. 10.1038/mto.2016.6 27162934 PMC4849432

[7] Jan M Scarfò I Larson RC Walker A Schmidts A Guirguis AA. Reversible ON- and OFF-Switch Chimeric Antigen Receptors Controlled by Lenalidomide. Sci Trans Med (2021) 13:575. 10.1126/scitranslmed.abb6295 PMC8045771 33408186

[8] Hoyos V Savoldo B Quintarelli C Mahendravada A Zhang M Vera J. Engineering CD19-Specific T Lymphocytes With interleukin-15 and a Suicide Gene to Enhance Their Anti-Lymphoma/Leukemia Effects and Safety. Leukemia (2010) 24(6):1160–70. 10.1038/leu.2010.75 PMC2888148 20428207

[9] Newick K O’Brien S Moon E Albelda SM. Car T Cell Therapy for Solid Tumors. Annu Rev Med (2017) 68:139–52. 10.1146/annurev-med-062315-120245 27860544

[10] Drent E Themeli M Poels R de Jong-Korlaar R Yuan H de Bruijn J. A Rational Strategy for Reducing On-Target Off-Tumor Effects of CD38-Chimeric Antigen Receptors by Affinity Optimization. Mol Ther (2017) 25(8):1946–58. 10.1016/j.ymthe.2017.04.024 PMC5542711 28506593

[11] Labanieh L Majzner RG Mackall CL. Programming CAR-T Cells to Kill Cancer. Nat BioMed Eng (2018) 2(6):377–91. 10.1038/s41551-018-0235-9 31011197

[12] Gordon WR Zimmerman B He L Miles LJ Huang J Tiyanont K. Mechanical Allostery: Evidence for a Force Requirement in the Proteolytic Activation of Notch. Dev Cell (2015) 33(6):729–36. 10.1016/j.devcel.2015.05.004 PMC4481192 26051539

[13] Yang ZJ Yu ZY Cai YM Du RR Cai L. Engineering of an Enhanced Synthetic Notch Receptor by Reducing Ligand-Independent Activation. Commun Biol (2020) 3(1):116. 10.1038/s42003-020-0848-x 32170210 PMC7069970

[14] Roybal KT Rupp LJ Morsut L Walker WJ McNally KA Park JS. Precision Tumor Recognition by T Cells With Combinatorial Antigen-Sensing Circuits. Cell (2016) 164(4):770–9. 10.1016/j.cell.2016.01.011 PMC4752902 26830879

[15] Srivastava S Salter AI Liggitt D Yechan-Gunja S Sarvothama M Cooper K. Logic-Gated ROR1 Chimeric Antigen Receptor Expression Rescues T Cell-Mediated Toxicity to Normal Tissues and Enables Selective Tumor Targeting. Cancer Cell (2019) 35(3):489–503.e8. 10.1016/j.ccell.2019.02.003 30889382 PMC6450658

[16] Ruella M Barrett DM Kenderian SS Shestova O Hofmann TJ Perazzelli J. Dual CD19 and CD123 Targeting Prevents Antigen-Loss Relapses After CD19-directed Immunotherapies. J Clin Invest (2016) 126(10):3814–26. 10.1172/jci87366 PMC5096828 27571406

[17] Bielamowicz K Fousek K Byrd TT Samaha H Mukherjee M Aware N. Trivalent CAR T Cells Overcome Interpatient Antigenic Variability in Glioblastoma. Neuro Oncol (2018) 20(4):506–18. 10.1093/neuonc/nox182 PMC5909636 29016929

[18] Kulemzin SV Kuznetsova VV Mamonkin M Taranin AV Gorchakov AA. [Car T-Cell Therapy: Balance of Efficacy and Safety]. Mol Biol (Mosk) (2017) 51(2):274–87. 10.7868/s0026898417020148 28537234

[19] D’Aloia MM Zizzari IG Sacchetti B Pierelli L Alimandi M. CAR-T Cells: The Long and Winding Road to Solid Tumors. Cell Death Dis (2018) 9(3):282. 10.1038/s41419-018-0278-6 29449531 PMC5833816

[20] Nurmik M Ullmann P Rodriguez F Haan S Letellier E. In Search of Definitions: Cancer-Associated Fibroblasts and Their Markers. Int J Cancer (2020) 146(4):895–905. 10.1002/ijc.32193 30734283 PMC6972582

[21] Wang LC Lo A Scholler J Sun J Majumdar RS Kapoor V. Targeting Fibroblast Activation Protein in Tumor Stroma With Chimeric Antigen Receptor T Cells Can Inhibit Tumor Growth and Augment Host Immunity Without Severe Toxicity. Cancer Immunol Res (2014) 2(2):154–66. 10.1158/2326-6066.cir-13-0027 PMC4007316 24778279

[22] Smits NC Sentman CL. Bispecific T-Cell Engagers (BiTEs) as Treatment of B-Cell Lymphoma. J Clin Oncol (2016) 34(10):1131–3. 10.1200/jco.2015.64.9970 PMC5085271 26884583

[23] Huehls AM Coupet TA Sentman CL. Bispecific T-Cell Engagers for Cancer Immunotherapy. Immunol Cell Biol (2015) 93(3):290–6. 10.1038/icb.2014.93 PMC4445461 25367186

[24] Ellerman D. Bispecific T-Cell Engagers: Towards Understanding Variables Influencing the *In Vitro* Potency and Tumor Selectivity and Their Modulation to Enhance Their Efficacy and Safety. Methods (2019) 154:102–17. 10.1016/j.ymeth.2018.10.026 30395966

[25] de Sostoa J Fajardo CA Moreno R Ramos MD Farrera-Sal M Alemany R. Targeting the Tumor Stroma With an Oncolytic Adenovirus Secreting a Fibroblast Activation Protein-Targeted Bispecific T-Cell Engager. J Immunother Cancer (2019) 7(1):19. 10.1186/s40425-019-0505-4 30683154 PMC6347837

[26] Choi BD Yu X Castano AP Bouffard AA Schmidts A Larson RC. CAR-T Cells Secreting BiTEs Circumvent Antigen Escape Without Detectable Toxicity. Nat Biotechnol (2019) 37(9):1049–58. 10.1038/s41587-019-0192-1 31332324

[27] O’Shea JJ Schwartz DM Villarino AV Gadina M McInnes IB Laurence A. The JAK-STAT Pathway: Impact on Human Disease and Therapeutic Intervention. Annu Rev Med (2015) 66:311–28. 10.1146/annurev-med-051113-024537 PMC5634336 25587654

[28] Kagoya Y Tanaka S Guo T Anczurowski M Wang CH Saso K. A Novel Chimeric Antigen Receptor Containing a JAK-STAT Signaling Domain Mediates Superior Antitumor Effects. Nat Med (2018) 24(3):352–9. 10.1038/nm.4478 PMC5839992 29400710

[29] Goswami R Kaplan MH. STAT Transcription Factors in T Cell Control of Health and Disease. Int Rev Cell Mol Biol (2017) 331:123–80. 10.1016/bs.ircmb.2016.09.012 28325211

[30] Schneider D Xiong Y Wu D Nlle V Schmitz S Haso W. A Tandem CD19/CD20 CAR Lentiviral Vector Drives On-Target and Off-Target Antigen Modulation in Leukemia Cell Lines. J Immunother Cancer (2017) 5:42. 10.1186/s40425-017-0246-1 28515942 PMC5433150

[31] Roybal KT Williams JZ Morsut L Rupp LJ Kolinko I Choe JH. Engineering T Cells With Customized Therapeutic Response Programs Using Synthetic Notch Receptors. Cell (2016) 167(2):419–32.e16. 10.1016/j.cell.2016.09.011 27693353 PMC5072533

[32] Rosewell Shaw A Porter CE Watanabe N Tanoue K Sikora A Gottschalk S. Adenovirotherapy Delivering Cytokine and Checkpoint Inhibitor Augments Car T Cells Against Metastatic Head and Neck Cancer. Mol Ther (2017) 25(11):2440–51. 10.1016/j.ymthe.2017.09.010 PMC5675597 28974431

[33] Yang M Wang L Ni M Schubert M-L Neuber B Hückelhoven-Krauss A. The Effect of Apoptosis Inhibitor Blockade Agents on the Third Generation CD19 Car T Cells. Blood (2019) 134:5620–. 10.1182/blood-2019-125622

[34] Chen L Qiu X Wang X He J. FAP Positive Fibroblasts Induce Immune Checkpoint Blockade Resistance in Colorectal Cancer *Via* Promoting Immunosuppression. Biochem Biophys Res Commun (2017) 487(1):8–14. 10.1016/j.bbrc.2017.03.039 28302482

[35] Floss DM Klöcker T Schröder J Lamertz L Mrotzek S Strobl B. Defining the Functional Binding Sites of Interleukin 12 Receptor β1 and Interleukin 23 Receptor to Janus Kinases. Mol Biol Cell (2016) 27(14):2301–16. 10.1091/mbc.E14-12-1645 PMC4945146 27193299

[36] Morris R Kershaw NJ Babon JJ. The Molecular Details of Cytokine Signaling Via the JAK/STAT Pathway. Protein Sci (2018) 27(12):1984–2009. 10.1002/pro.3519 30267440 PMC6237706

[37] Yager N Haddadeen C Powell M Payne A Allen R Healy E. Expression of PI3K Signaling Associated With T Cells in Psoriasis is Inhibited by Seletalisib, a PI3Kdelta Inhibitor, and Is Required for Functional Activity. J Invest Dermatol (2018) 138(6):1435–9. 10.1016/j.jid.2017.12.028 29307594

[38] Li Q Eppolito C Odunsi K Shrikant PA. IL-12-Programmed Long-Term CD8+ T Cell Responses Require STAT4. J Immunol (Baltimore Md 1950) (2006) 177(11):7618–25. 10.4049/jimmunol.177.11.7618 17114431

[39] Eshima K Misawa K Ohashi C Iwabuchi K. Role of T-bet, the Master Regulator of Th1 Cells, in the Cytotoxicity of Murine CD4(+) T Cells. Microbiol Immunol (2018) 62:348–56. 10.1111/1348-0421.12586 29577371

[40] Luo H Wu X Sun R Su J Wang Y Dong Y. Target-Dependent Expression of IL12 by SynNotch Receptor-Engineered NK92 Cells Increases the Antitumor Activities of CAR-T Cells. Front Oncol (2019) 9:1448. 10.3389/fonc.2019.01448 31921693 PMC6930917

[41] Wei L Vahedi G Sun HW Watford WT Takatori H Ramos HL. Discrete Roles of STAT4 and STAT6 Transcription Factors in Tuning Epigenetic Modifications and Transcription During T Helper Cell Differentiation. Immunity (2010) 32(6):840–51. 10.1016/j.immuni.2010.06.003 PMC2904651 20620946

[42] Yu Y Xiao CH Tan LD Wang QS Li XQ Feng YM. Cancer-Associated Fibroblasts Induce Epithelial-Mesenchymal Transition of Breast Cancer Cells Through Paracrine TGF-beta Signalling. Br J Cancer (2014) 110(3):724–32. 10.1038/bjc.2013.768 PMC3915130 24335925

[43] Sun Y Fan X Zhang Q Shi X Xu G Zou C. Cancer-Associated Fibroblasts Secrete FGF-1 to Promote Ovarian Proliferation, Migration, and Invasion Through the Activation of FGF-1/FGFR4 Signaling. Tumour Biol (2017) 39(7):1010428317712592. 10.1177/1010428317712592 28718374

[44] Ratajczak-Wielgomas K Grzegrzolka J Piotrowska A Gomulkiewicz A Witkiewicz W Dziegiel P. Periostin Expression in Cancer-Associated Fibroblasts of Invasive Ductal Breast Carcinoma. Oncol Rep (2016) 36(5):2745–54. 10.3892/or.2016.5095 27633896

[45] Ni WD Yang ZT Cui CA Cui Y Fang LY Xuan YH. Tenascin-C is a Potential Cancer-Associated Fibroblasts Marker and Predicts Poor Prognosis in Prostate Cancer. Biochem Biophys Res Commun (2017) 486:607–12. 10.1016/j.bbrc.2017.03.021 28341124

[46] Niknejad H Khayat-Khoei M Peirovi H. Inhibition of MMPs Might Increase Anticancer Properties of Amniotic Epithelial Cells. Med Hypotheses (2012) 78(5):690–1. 10.1016/j.mehy.2012.02.014 22401776

[47] Liu T Han C Wang S Fang P Ma Z Xu L. Cancer-Associated Fibroblasts: An Emerging Target of Anti-Cancer Immunotherapy. J Hematol Oncol (2019) 12(1):86. 10.1186/s13045-019-0770-1 31462327 PMC6714445

[48] Niknejad H Yazdanpanah G Ahmadiani A. Induction of Apoptosis, Stimulation of Cell-Cycle Arrest and Inhibition of Angiogenesis Make Human Amnion-Derived Cells Promising Sources for Cell Therapy of Cancer. Cell Tissue Res (2016) 363(3):599–608. 10.1007/s00441-016-2364-3 26846225

[49] LeBleu VS Kalluri R. A Peek Into Cancer-Associated Fibroblasts: Origins, Functions and Translational Impact. Dis Models Mech (2018) 11(4):dmm029447. 10.1242/dmm.029447 PMC5963854 29686035

[50] Freedman JD Duffy MR Lei-Rossmann J Muntzer A Scott EM Hagel J. An Oncolytic Virus Expressing A T-Cell Engager Simultaneously Targets Cancer and Immunosuppressive Stromal Cells. Cancer Res (2018) 78(24):6852–65. 10.1158/0008-5472.can-18-1750 30449733

[51] Wu L Wei Q Brzostek J Gascoigne NRJ. Signaling From T Cell Receptors (TCRs) and Chimeric Antigen Receptors (CARs) on T Cells. Cell Mol Immunol (2020) 17(6):600–12. 10.1038/s41423-020-0470-3 PMC7264185 32451454

[52] Watanabe K Kuramitsu S Posey AD Jr June CH. Expanding the Therapeutic Window for CAR T Cell Therapy in Solid Tumors: The Knowns and Unknowns of CAR T Cell Biology. Front Immunol (2018) 9:2486. 10.3389/fimmu.2018.02486 30416506 PMC6212550

[53] Stenger D Stief TA Kaeuferle T Willier S Rataj F Schober K. Endogenous TCR Promotes *In Vivo* Persistence of CD19-CAR-T Cells Compared to a CRISPR/Cas9-Mediated TCR Knockout CAR. Blood (2020) 136(12):1407–18. 10.1182/blood.2020005185 PMC7612202 32483603

[54] Ham S Lima LG Lek E Möller A. The Impact of the Cancer Microenvironment on Macrophage Phenotypes. Front Immunol (2020) 11:1308. 10.3389/fimmu.2020.01308 32655574 PMC7324670

[55] Jacobsen T Yi G Al Asafen H Jermusyk AA Beisel CL Reeves GT. Tunable Self-Cleaving Ribozymes for Modulating Gene Expression in Eukaryotic Systems. PloS One (2020) 15(4):e0232046. 10.1371/journal.pone.0232046 32352996 PMC7192461

[56] Lienert F Lohmueller JJ Garg A Silver PA. Synthetic Biology in Mammalian Cells: Next Generation Research Tools and Therapeutics. Nat Rev Mol Cell Biol (2014) 15(2):95–107. 10.1038/nrm3738 24434884 PMC4032074

